# The role of presynaptic dopaminergic imaging in acquired neurological conditions affecting basal ganglia: a systematic review

**DOI:** 10.3389/fneur.2025.1660747

**Published:** 2025-11-04

**Authors:** Elena Ardila Jurado, Lisa Zünd-Hofer, Florian Brugger, Nicolas Nicastro, Kailash P. Bhatia, Georg Kägi

**Affiliations:** ^1^Department of Neurology, Cantonal Hospital, St. Gallen, Switzerland; ^2^Department of Clinical Neurosciences, Geneva University Hospitals, Geneva, Switzerland; ^3^Department of Clinical and Movement Neurosciences, UCL, Queen Square, Institute of Neurology, University College London, London, United Kingdom; ^4^Department of Neurology, Inselspital, University Hospital Bern, Bern, Switzerland

**Keywords:** DAT SPECT, dopaminergic imaging, SPECT, PET, normal pressure hydrocephalus, Holmes Tremor, vascular parkinsonism, secondary parkinsonism

## Abstract

**Background:**

Dopaminergic imaging has become a pivotal tool in the diagnosis of Parkinson's disease (PD) and related disorders. Its ability to assess presynaptic dopamine transporter function provides crucial insights for distinguishing PD from other acquired neurological disorders. Recent advances have also demonstrated its utility in evaluating diseases beyond PD, including non-degenerative conditions associated with parkinsonism.

**Objectives:**

This review aims to explore the diagnostic and therapeutic value of dopaminergic imaging across a range of acquired disorders, including normal pressure hydrocephalus, Holmes tremor, vascular parkinsonism, infectious and metabolic diseases as well as autoimmune encephalopathies with a particular focus on its clinical implications, imaging patterns and predictive value for treatment response. We excluded drug induced conditions as they have been extensively reviewed previously.

**Methods:**

A comprehensive systematic literature search focusing on studies that utilized dopaminergic imaging techniques was conducted in PubMed. We used the terms “DaTScan.” “Dopaminergic imaging,” “dopamine transporter,” “Single-photon emission computed tomography,” “FP CIT 123 SPECT,” “^123^I-ioflupane,” “TRODAT” and “^18^F-DOPA” and focused on acquired neurological disorders. Diagnostic accuracy and imaging patterns across these conditions were analyzed.

**Results:**

Dopaminergic imaging revealed variable deficits across acquired disorders, with distinct patterns aiding in the differential diagnosis. In normal pressure hydrocephalus, imaging often shows a reduction in striatal dopamine transporter binding which was reversed post-shunt surgery, distinguishing it from neurodegenerative parkinsonisms. In Holmes tremor, significant presynaptic dopaminergic deficits were associated with levodopa responsiveness. Vascular parkinsonism exhibited more diffuse and symmetric dopamine transporter reductions compared to idiopathic PD, correlating with poorer levodopa response.

**Conclusion:**

Dopaminergic imaging plays a crucial role in differentiating PD from acquired diseases presenting with parkinsonism. Its diagnostic utility, combined with clinical and pathological findings, enhances therapeutic decision-making, particularly in more common conditions like normal pressure hydrocephalus, Holmes tremor and vascular parkinsonism. As imaging techniques continue to evolve, their integration into clinical practice will further support personalized treatment strategies.

## Introduction

The dopaminergic system plays a central role in various neurological and psychiatric functions, including motor control, cognition, executive functions, reward processing, motivation, and neuroendocrine regulation. This system operates through three distinct pathways: the nigrostriatal pathway, originating in the substantia nigra and innervating the dorsal striatum (caudate nucleus and putamen); the mesolimbic and mesocortical pathways, extending from the ventral tegmental area to limbic regions (ventral striatum) and the prefrontal cortex; and the tuberoinfundibular pathway, connecting the hypothalamic arcuate nucleus to the pituitary gland, which regulates neuroendocrine activity (1, 2). Dopamine (DA) is rapidly cleared from the synaptic cleft via reuptake by the dopamine transporter (DAT) on the presynaptic membrane. DAT-mediated reuptake is crucial for terminating dopaminergic signaling and maintaining synaptic homeostasis (2–4).

Dopaminergic imaging techniques, including DAT imaging via single-photon emission computed tomography (SPECT) and radiotracers such as ^123^I-ioflupane (DaTscan^®^, also known as ^123^I-FP-CIT), ^123^I-β-CIT (5), ^123^I-IPT (6), and TRODAT (7) among others, allow for the quantification of presynaptic dopaminergic function by measuring the density and activity of DAT (4, 8, 9). Other positron emission tomography (PET) tracers, such as ^18^F-DOPA (fluorine-18 3,4-dihydroxyphenylalanine), converted by L-amino acid decarboxylase (AAAD) to fluorodopamine, assess terminal dopa decarboxylase activity and dopamine turnover (10), while markers for vesicular monoamine transporter 2 (VMAT2) and postsynaptic dopamine receptors offer insight into dopamine storage and receptor function, respectively (3).

Dopaminergic imaging has established its significance in the accurate diagnosis of Parkinson's Disease (PD). By visualizing DAT density in the striatum, dopaminergic imaging enables clinicians to differentiate PD from other conditions with similar clinical presentations, such as essential tremor and drug-induced parkinsonism (DIP), where DAT binding is preserved. While some patients with DIP recover upon withdrawal of the offending drug, others continue to experience motor symptoms, raising concerns about an underlying neurodegenerative disorder (11). In such cases, dopaminergic imaging plays a crucial role in distinguishing DIP from degenerative parkinsonism, aiding in clinical decision-making. Studies have demonstrated a sensitivity and specificity exceeding 80% for differentiating DIP from PD, reinforcing its diagnostic value (12). Although DIP, like essential tremor, remains an important differential diagnosis, both conditions have been extensively studied, and a comprehensive discussion of these entities exceeds the scope of this review.

Several landmark studies have reinforced the accuracy of imaging in the diagnostic process. A neuropathological cohort study by Hastings et al. (13) demonstrated that dopaminergic imaging achieves a sensitivity of 100% for PD. The diagnostic precision of dopaminergic imaging is substantiated by additional neuropathological studies that revealed a high correlation between *in vivo* dopaminergic imaging results and the extent of dopaminergic neuron loss in the substantia nigra, confirming the validity of dopamine transporter imaging as an excellent *in vivo* marker of nigrostriatal dopaminergic degeneration (14, 15). The Movement Disorder Society's (MDS) 2015 diagnostic criteria for PD emphasized this by including normal functional neuroimaging of the presynaptic dopaminergic system as an exclusion criterion for the diagnosis of PD (16). The critical role of neuroimaging in accurately diagnosing PD has also been emphasized in a position paper from the movement disorder society published in 2023 (17).

Beyond its role in diagnosing PD, dopaminergic imaging is increasingly recognized for its utility in a variety of other acquired neurological disorders. Conditions such as normal pressure hydrocephalus, Holmes Tremor, and manganism have some degree of dopaminergic dysfunction and thus might exhibit abnormal dopaminergic imaging.

This review aims to explore the role of dopaminergic imaging beyond accepted indications and across various acquired diseases, highlighting its diagnostic value and implications for patient care. This imaging modality not only enhances diagnostic accuracy but also serves as a critical tool for therapeutic decision-making.

## Methods

In order to study non-degenerative conditions associated with parkinsonism, we undertook a comprehensive systematic search of literature published in PubMed from database inception to 15th of march 2024, with no time restrictions. We used the terms “DaTSCAN,” “Dopaminergic imaging,” “dopamine transporter,” “Single-photon emission computed tomography,” “FP CIT 123 SPECT,” “^123^I-ioflupane,” “TRODAT” and “^18^F-DOPA” combined with conditions like “Normal pressure hydrocephalus,” “Holmes Tremor,” “vascular parkinsonism,” “AV fistula,” “toxoplasmosis,” “HIV associated parkinsonism,” “SARS-CoV-2,” “Covid 19,” “metabolic disease,” “diabetic uremic syndrome,” “liver cirrhosis”, “manganism,” “osmotic demyelination syndrome,” “secondary chorea,” “subacute sclerosing panencephalitis,” “autoimmune encephalitis,” “anti-igLON5 disease” and “Creutzfeld Jakob disease.”

Studies were included if they reported presynaptic dopaminergic imaging on acquired neurological conditions affecting the basal ganglia. SPECT Studies with different radiotracers such as ^123^I-FP-CIT (DaTSCAN^®^), ^123^I-β-CIT, ^123^I-IPT, TRODAT, as well as ^18^F-DOPA PET studies were included, without restriction on the assessment used (visual vs. semiquantitative vs. quantitative). Articles in English, German, and Spanish were screened in full and included when they met eligibility criteria. Studies were excluded if they were limited to postsynaptic dopaminergic imaging.

The PubMed search yielded 873 records. After title and abstract screening, 148 articles were selected for full-text review. Following the application of inclusion and exclusion criteria, 95 studies reporting original presynaptic dopaminergic imaging findings in acquired neurological conditions were included in the final synthesis. Studies that provided only background information without original imaging results were excluded from this count.

Study selection was performed by a single reviewer. Given the rarity of some of the included conditions and the predominance of case reports and small case series, no formal risk-of-bias was performed. The review was not pre-registered.

Details of assessment of presynaptic dopaminergic imaging are summarized in Box 1.

Box 1 **Assessment of presynaptic dopaminergic imaging.**

Assessment of dopaminergic imaging can be performed through several methods, each with its strengths and limitations. Visual evaluation, the most widely used method, is performed by a trained nuclear medicine physician or radiologist and judges shape, extent, symmetry and intensity of striatal uptake (3). While quick and cost-effective, this method is subject to significant inter- and intra-observer variability (183), underlying the rater's experience. Clinically, visual evaluation is often sufficient because symptoms typically emerge only after a significant proportion of striatal synapses have degenerated.

Semiquantitative analysis combines visual interpretation with standardized techniques to quantify tracer uptake, providing more objective data and allowing for longitudinal or between patient comparisons. The striatal binding ratio (SBR) is calculated as (3):

SBR = [mean counts of striatal ROI (Region of interest)–mean counts of background ROI]/mean counts of background ROI

Those analysis are defined as the ratio of activity in the striatum (8), compared to activity in a reference region with low DAT concentration, normally the occipital area. Limitations include interobserver variation and errors in ROI placement (183). A discrepancy between visual and automated analysis has been observed in 10% of cases, especially in older patients (184). Comparison with age-matched reference values is crucial for accurate interpretation and validity. A study by Nicastro et al. (186) established site-specific age-related reference values, noting a significant negative linear effect of age on DAT uptake with a decline of ~6.8% per decade (186). Quantitative assessments, using advanced imaging analysis techniques and algorithms, offer highly objective and reproducible evaluations but are more suited for research due to their complexity and the need for specialized software and expertise.

Efforts in recent years have focused on improving dopaminergic imaging techniques and results. Advanced methods, such as automated semi-quantitative analyses and the development of specialized software tools like BRASS and DaTQUANT (185), aim to enhance diagnostic accuracy and reduce variability (183, 187, 188).

## Idiopathic normal pressure hydrocephalus

The exact pathophysiological link between Parkinsonism and idiopathic normal pressure hydrocephalus (iNPH) remains unclear. It is hypothesized that the abnormal pulsation of cerebrospinal fluid (CSF) in iNPH may cause secondary impairment to the nigrostriatal dopaminergic pathway in the striatum (18, 19). Hydrocephalus might directly impact the caudate nucleus due to its proximity to the lateral ventricles, however this does not explain putaminal dysfunction (18). Additionally, severe and longstanding cases of iNPH might injure the meso-limbic dopaminergic pathway and the ascending reticular activating system (20).

However, the etiology of iNPH remains debated: is it primarily a CSF-dynamics disorder with secondary parenchymal damage, or can ventriculomegaly in late adulthood represent an early manifestation of an underlying neurodegenerative disorder? Recent work suggests that ventriculomegaly in late adulthood often coincides with progressive neurodegeneration rather than a purely mechanical process (21). Neuropathological studies have found that patients initially diagnosed with iNPH may later develop Alzheimer's disease (AD), Lewy body dementia (LBD), or progressive supranuclear palsy (PSP), raising the possibility that a subset of iNPH cases might represent an early “neurodegenerative NPH” rather than a separate entity (22, 23). Consistent with this overlap, Sakurai et al (24) using CSF real-time quaking induced conversion (RT-QuIC) assays for alpha-synuclein aggregation found a co-prevalence of 29.1% of iNPH with PD and PD dementia (PDD) and 10.1% of iNPH and LBD. Further, other authors have highlighted the complexity of its pathophysiology and its overlap with PD and atypical parkinsonian syndromes (APS), particularly PSP and LBD (25). This distinction is crucial, as it influences both diagnostic approaches and treatment expectations.

Against this background, presynaptic dopaminergic imaging plays an essential role in distinguishing between reversible parkinsonism due to mechanical factors and irreversible neurodegeneration (21).

Studies investigating DAT imaging in iNPH report heterogeneous results (see Table 1). While Ouchi et al. (19) found preserved presynaptic activity in 8 iNPH patients, larger series and a case report have reported reduced DAT density with a prevalence varying from 31 to 62% of iNPH patients (26–29), or even up to 91% in one case series (30). However, the reduction in SBR uptake seems more prominent in PD, PDD and LBD compared to iNPH (24).

**Table 1:** Summary of dopaminergic imaging studies in iNPH.

**Authors and year of publication**	**Nr patients**	**Radiotracer**	**Finding in dopaminergic imaging**	**Conclusion**
Allali et al. (2018)	56 subjects: 26 iNPH, and 30 iNPH mimics	^123^I-FP-CIT	Semi-quantitative assessment distinguished iNPH from mimics; iNPH showed less frequent DAT reduction: normal SBR in 69.2% of iNPH and 37.9% of mimics (*p* = 0.02)	iNPH patients exhibit distinct dopaminergic patterns, improving patient selection for surgery
Broggi et al. (2016)	30	^123^I-FP-CIT	Striatal dopaminergic deficit identified in 46.5% of iNPH patients.	VP shunt combined with dopamine therapy improved outcomes in iNPH patients with pathological dopaminergic imaging
Chang et al. (2020)	39	^99^mTc-TRODAT-1	19 Patients showed no abnormality in the dopaminergic imaging, while 20 Patients showed different levels of dopaminergic deficit	Dopaminergic degeneration comorbidity neutralized the degree of improvement after surgery
Del Gamba et al. (2020)	1	^123^I-FP-CIT	Abnormal asymmetrical uptake partially reversible post-VP shunt	DAT abnormalities may reflect reversible mechanical compression rather than degeneration
Kinugawa et al. (2009)	1	^123^I-FP-CIT	Moderate reduction in striatal uptake with a similar symmetrical decrease in caudate and putamen	The pattern of ^123^I-FP-CIT uptake, featuring a similar symmetrical decrease in the caudate and putamen, differed from that seen in neurodegenerative PD, where denervation is usually asymmetrical and posterior-to-anterior. Reversion of the parkinsonism after third ventriculostomy was associated with improved ^123^I-FP-CIT uptake
Lee et al. (2020)	11	^18^F-FP-CIT	Striatal dopaminergic depletion found in 90.9% of iNPH patients, with predominant caudate nucleus involvement	Pattern of DAT loss in iNPH differs from degenerative parkinsonian disorders; highlights diagnostic potential
Ouchi et al. (2006)	8 iNPH, 8 HC	^11^C-CFT	Preserved presynaptic DAT binding	No correlation between BPs and morphometric measures
Pozzi et al. (2020)	50 iNPH, 25 PD, 40 HC	^123^I-FP-CIT	62% of patients with iNPH showed a reduced SBR. iNPH showed symmetric caudate-dominant DAT deficits, differing from PD pattern	DAT deficits in iNPH correlate with parkinsonism severity; imaging differentiates iNPH from PD
Racette et al. (2004)	1	^18^F-DOPA	Reduced uptake in the caudate and putamen with relative sparing of the posterior putamen	The ratio of caudate to posterior putamen Ki was below the range found in the idiopathic PD controls
Sakurai et al. (2021)	79	^123^I-FP-CIT	DAT reduction was more severe in iNPH with comorbid synucleinopathies	Synucleinopathies coexist with iNPH. These can be differentiated by performing DAT SPECT and RT-QuIC
Todisco et al. (2021)	92	^123^I-FP-CIT	Gait impairment correlated with caudate DAT density. Locomotor phenotype showed lower striatal DAT density compare to disequilibrium phenotype	Shunt surgery improved striatal DAT binding and gait in iNPH patients, highlightening the reversibility of striatal dysfunction in iNPH

This table provides an overview of studies investigating dopaminergic imaging in patients with iNPH. For each study, the authors, year of publication, number of patients, radiotracer, key imaging findings and main conclusions are summarized.

**Figure 1:**
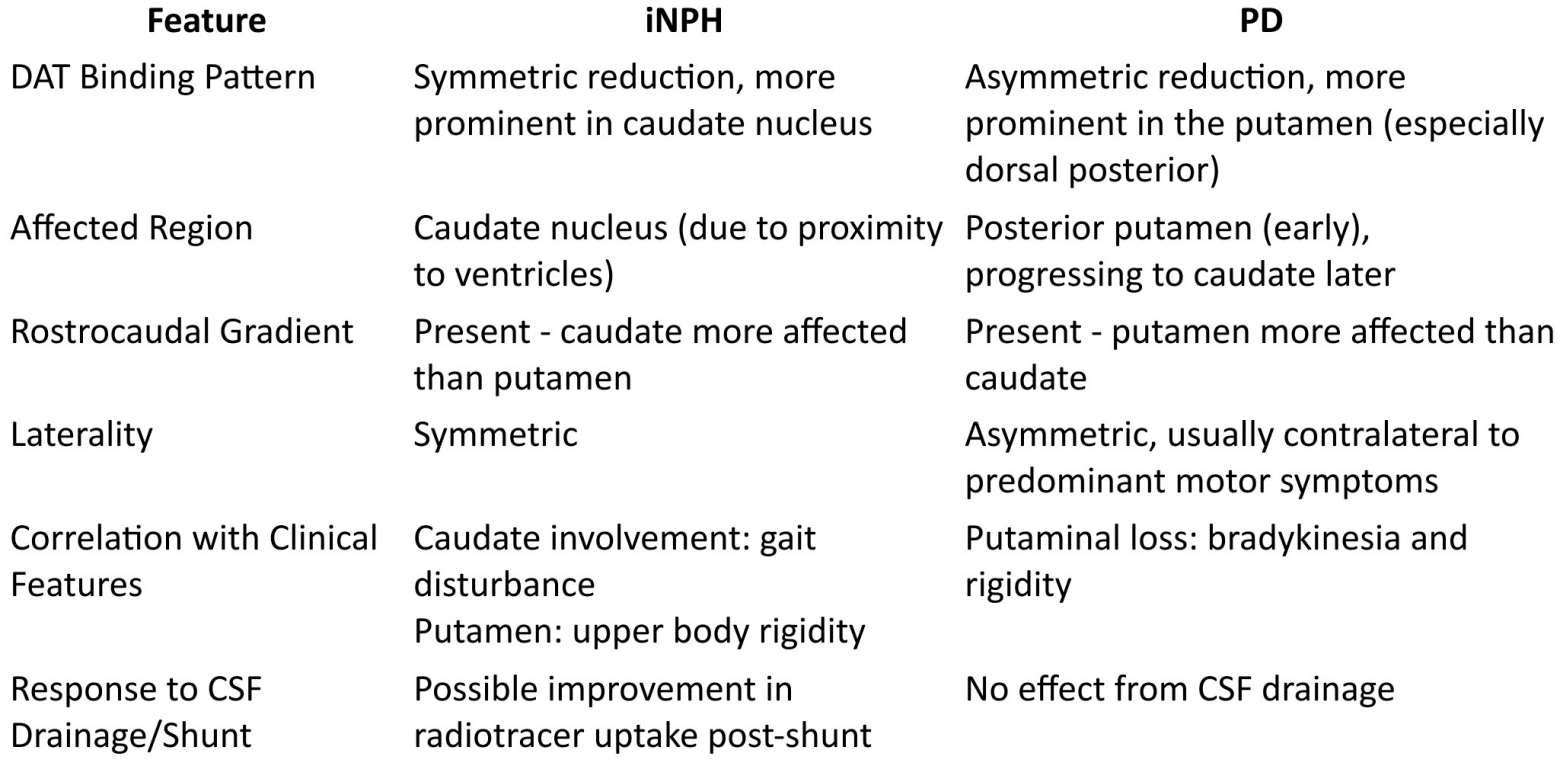
A schematic comparison of dopaminergic imaging findings in patients with iNPH and PD. Key features such as DAT binding patterns, affected regions, rostrocaudal gradient, laterality, correlation with clinical symptoms, and response to CSF drainage are summarized.

iNPH shows a distinct pattern of dopaminergic depletion, characterized by a prominent caudate nucleus impairment and a rostrocaudal gradient, differing from the pattern observed in PD, which tends to primarily affect dorsal posterior putamen with relatively preserved caudate nucleus (18, 30). The striatal pattern is typically more symmetric in iNPH compared to the lateralized deficits found primarily in PD (28, 30–32). The caudate/putamen (C/P) ratio distinguished iNPH from PD with high specificity and sensitivity, highlighting the distinct dopaminergic depletion pattern in iNPH (Figure 1). The emphasis on the caudate nucleus may reflect its greater vulnerability due to its proximity to dilated ventricles. It has been proposed that lower putaminal DAT density is linked to the presence and severity of upper-body parkinsonism, whereas caudate dysfunction is linked to more severe gait disturbances (31).

A notable clinical distinction from PD is that parkinsonism in iNPH may improve, at least temporarily, after CSF drainage or shunt surgery (25, 29, 31, 33). Post-surgery improvements in gait accompanied by increases in caudate DAT binding have been observed, suggesting reversible presynaptic dopaminergic dysfunction in iNPH attributable to mechanical compression rather than neurodegeneration (31). However, long-term follow-up studies challenge this notion: many patients initially diagnosed with iNPH who exhibit only transient improvements post-shunting later progress to neurodegenerative disorders such as AD, PSP, or LBD, implying that a subset may be a preclinical stage of neurodegeneration rather than a purely mechanical disorder (21, 23). This may help explain poorer surgical outcome in patients with impaired dopaminergic imaging (26, 27, 33). Evidence is not uniform, though: Broggi et al. (27) reported favorable outcomes in six patients with pathological presynaptic dopaminergic imaging achieving >30% motor Unified Parkinson Disease Rating Scale (UPDRS) improvement regardless of whether they received ventriculoperitoneal shunt (VPS) alone or with additional dopaminergic therapy.

There are some discordant findings regarding dopa-responsiveness in patients with iNPH. While some authors describe an improvement of parkinsonian symptoms under dopaminergic therapy (18, 32), other authors have not observed any improvement after dopamine therapy, pointing to a possible involvement of the postsynaptic dopaminergic system (31).

In summary, dopaminergic imaging can help differentiate iNPH from degenerative Parkinsonism by highlighting distinct patterns, such as a milder symmetric caudate-dominant depletion in iNPH vs. asymmetric putaminal-dominant loss in PD (Figure 1). However, overlap remains, and accumulating evidence suggests that iNPH itself may not always be a separate entity but, in some cases, a precursor to neurodegeneration (21, 23). This highlights the need for a more nuanced diagnostic approach.

## Holmes Tremor

Holmes Tremor (HT) is a low frequency (< 5 Hz) rest, postural and intention tremor. The impairment of the dopaminergic nigrostriatal system is probably responsible for the rest tremor; and the impairment of the cerebello-thalamo-cortical or dentato-rubro-olivary pathways for the postural and intentional tremor components (34, 35). The latency from lesion to tremor onset is variable, ranging from 2 weeks to 2 years (36).

The etiologies of this tremor vary widely and so do the associated symptoms. Raina et al. (36) studied 29 HT patients, finding the most common causes to be vascular (48%) and traumatic (17%). Other causes included demyelinating diseases, AIDS-related infections, unruptured arterio-venous malformation (AVM), and metastatic cancer. HT occurs rarely isolated: common associated symptoms included hemiparesis (62%), ataxia (51%), hypoesthesia (27%), dystonia (24%) and dysarthria (24%). Cranial nerve involvement has been described in 24% of patients, with cranial nerve III being most commonly affected (36, 37). Dystonia and abnormal proprioception are often present when the underlying pathology is located in the thalamus (34). Less frequent co-features included vertical gaze disorders (6%), bradykinesia/rigidity (6%), myoclonus (3%), and seizures (3%) (36). However, the accompanying symptoms vary with lesion location and etiology.

Presynaptic dopaminergic imaging plays a critical role in diagnosing HT by revealing striatal dopaminergic denervation (Table 2). Many authors have described a markedly reduction of striatal dopamine uptake in HT due to different underlying etiologies: midbrain cavernoma (37–39), upper peduncular lesion (40), thalamic hemorrhage (41), radiation therapy-induced bleeding (42), arachnoid cyst (43), post-Epstein–Barr virus infection (38), midbrain stroke (44), posttraumatic lesion (45), thalamic/midbrain low-grade primary glioma (46). Although not all HT are related to presynaptic striatal denervation, if present, the degree of presynaptic denervation is usually more marked than in PD patients (40, 46).

**Table 2:** Summary of dopaminergic imaging studies in HT.

**Authors**	**Nr patients**	**Radiotracer**	**Finding in dopaminergic imaging**	**Conclusions**
Aubignat et al. (2023)	1	^123^I-FP-CIT	Major dopaminergic denervation on left side before arachnoid cyst surgery with complete normalization after arachnoid cyst fenestration	HT symptoms can resolve after surgical intervention for structural lesions
Gajos et al. (2017)	3	^123^I-FP-CIT	No significant asymmetry or abnormalities in presynaptic binding	Nigrostriatal deficit not always necessary; HT may involve multiple pathomechanisms
Gajos et al. (2010)	10	^123^I-FP-CIT	Minimal interhemispheric differences in DAT SPECT uptake; visual assessments showed no significant asymmetry	HT represents a heterogeneous spectrum of tremors with similar phenomenology but differing pathophysiology
Guedj et al. (2007)	1	^123^I-FP-CIT	Complete unilateral dopaminergic denervation in the affected hemisphere	Impairment of nigrostriatal pathways is critical in Holmes Tremor. Great improvement by stimulation of the VIM nucleus of the left thalamus
Hertel et al. (2006)	1	^123^I-Beta-CIT	Normal presynaptic dopamine reuptake binding	HT linked to mechanical and structural disruption; resolved after shunt placement
Juri et al. (2015)	1	^18^F-PR04.MZ	Significant striatal dopaminergic deficits detected with high-affinity ligand	Innovative tracers like ^18^F-PR04.MZ-PET provide precise imaging for diagnosing HT
Katschnig-Winter et al. (2015)	1	^123^I-FP-CIT	Nearly absent DAT binding in the ipsilateral striatum despite resolved midbrain lesions	DAT imaging predicts levodopa responsiveness in Holmes Tremor
Louarn et al. (2020)	1	^123^I-FP-CIT	Normal uptake in the contralateral striatum and denervation in the ipsilateral side	Nigrostriatal denervation due to midbrain lesion responsible for HT
Paviour et al. (2006)	1	^123^I-FP-CIT	Reduced tracer transport in the affected hemisphere, especially the putamen	Combined disruption of cerebello-rubro-thalamic and nigrostriatal pathways required for full syndrome
Remy et al. (1995)	6	^18^F-DOPA	Marked asymmetry in dopamine uptake, more pronounced denervation than in severe PD	Nigrostriatal dopaminergic system disruption is significant in peduncular tremors. All patients showed partial to complete improvement with levodopa
Schreuder et al. (2010)	1	^123^I-FP-CIT	Significant dopaminergic denervation in the putamen and caudate on the affected side	Midbrain lesions can cause dopaminergic deficits leading to HT
Seidel et al. (2009)	1	^123^I-Beta-CIT	Complete absence of dopamine transporter binding in the affected striatum	HT linked to nigrostriatal dopaminergic dysfunction and cerebellothalamic disruption
Sung et al. (2009)	1	^99^mTc-TRODAT-1	Bilateral reduction in tracer uptake with greater reduction on the affected side with partial improvement 3 years after onset of tremor	Involvement of both nigrostriatal and dentate-rubro-thalamic pathways contributes to HT. Regeneration of the nigrostriatal system is possible after the initial degeneration
Yen et al. (2022)	1	^123^IFP-CIT	Severe dopamine transporter deficiency on the affected side, being more severe than expected in PD	Dopaminergic imaging guides effective treatment with dopaminergic agents

This table summarizes published studies that investigate dopaminergic system involvement in patients with Holmes Tremor. For each study, the table lists the authors, year of publication, number of patients and tracer used, key findings in dopaminergic imaging and the study conclusions.

The nigrostriatal pathway is not always involved in the pathophysiology of HT as demonstrated by a few authors (Table 2). Gajos et al. described two case series with three and six HT patients, respectively, who exhibited no asymmetry in dopaminergic imaging. Neither the visual assessment nor the quantitative measurements showed any major asymmetry, except for 1 patient with a mild asymmetry in dopamine uptake and good response to levodopa treatment (47, 48). Hertel et al. (49) similarly described a case with a unilateral HT due to a midbrain lesion affecting the substantia nigra and cerebellothalamic pathway due to a communicating chronic hydrocephalus with normal presynaptic dopamine reuptake.

Due to the heterogeneous underlying pathophysiology of HT, therapeutic strategies vary. An important predictor for levodopa responsiveness is reduced DAT tracer binding on DAT imaging (38, 48, 50). There are multiple reports of an improvement of HT under dopaminergic therapy when the nigrostriatal pathway is affected (38, 40, 43, 46, 48, 50). Some authors describe an additional improvement of cognitive symptoms under dopaminergic therapy (46). Raina et al. (36) described an improvement under levodopa treatment in 54% of treated patients, with seven achieving near-complete control. Patients who responded positively to levodopa improved in the three components of HT. Unilateral thalamotomy provided excellent results in three patients (36). Nonetheless, exceptions occur: three case reports described a poor or no improvement under levodopa treatment beside markedly reduced striatal tracer transport imaging (37, 41, 44). Some patients with HT with a good response to levodopa develop significant and early levodopa induced dyskinesia, needing deep brain stimulation (DBS) to treat the symptoms (51). Guedj et al. (45) reported marked tremor control with left ventral intermediate nucleus (VIM) deep brain stimulation despite only partial levodopa response in a case with complete left dopaminergic denervation in SPECT imaging. Similarly, Hertel et al. (49) described a tremor improvement initially by CSF release after VPS placement and later with VIM-DBS in a patient with normal dopaminergic imaging.

**Figure 2:**
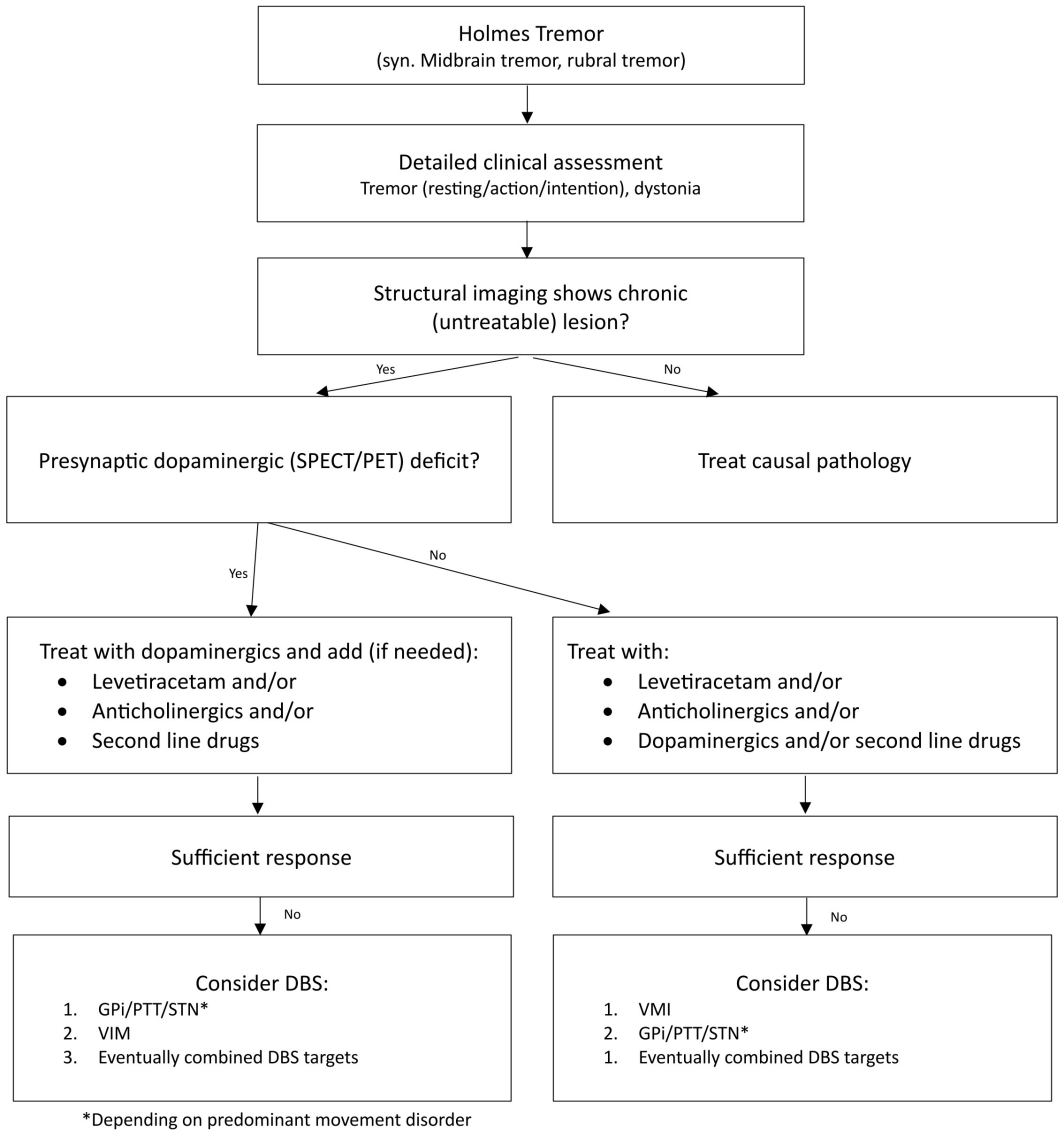
Treatment Algorithm for Patients with HT. This flowchart outlines the clinical assessment and treatment pathway for HT. Structural imaging evaluation to determine the presence of chronic, untreatable lesions. Use of presynaptic dopaminergic imaging to assess dopaminergic deficits. Pharmacological treatment options, including dopaminergics, anticholinergics, and second-line drugs based on the findings in dopaminergic imaging. In case of insufficient response to pharmacological treatment consider DBS.

Based on the literature and our experience, we propose the treatment algorithm shown in the Figure 2 for HT patients. Two cases have shown the reversibility of the symptoms as well as dopaminergic imaging findings after treating the underlying condition, suggesting potential regeneration of the nigrostriatal system (41). Aubignat documented a completely normalization of dopaminergic imaging after surgical intervention of the temporal arachnoid cyst that led to HT (43).

Overall, these studies and case reports underscore the critical role of dopaminergic imaging in diagnosing and understanding the pathophysiology of HT, particularly in assessing striatal dopaminergic denervation and predicting treatment response to dopaminergic agents (Table 2).

## Vascular disease

### Vascular parkinsonism

Vascular Parkinsonism (VP) is a clinical syndrome characterized by parkinsonian features associated with cerebrovascular disease, as highlighted in Zijlmans' diagnostic criteria (52). Two presentations are recognized: an acute onset (VPa) typically occurring within a year after a strategic infarct affecting the basal ganglia, thalamus, or frontal lobe, or a more insidious, progressive onset (VPi) on a background of diffuse subcortical white matter lesions, leading to symmetrical lower-body parkinsonism and cognitive dysfunction (52). Clinical clues favoring VP include stepwise progression (53), prominent gait impairment with leg predominant symptoms (52, 54–58) and co-occurrence of pyramidal signs, pseudobulbar palsy and urinary incontinence (52, 57, 59, 60). Furthermore, VP patients are generally older at the onset of motor symptoms (53, 56, 57, 61), have shorter disease duration compared to PD (56, 57, 61), and often present with more symmetric motor involvement (52, 59, 60). PD is suggested by pill rolling resting tremor (52, 56, 57), typical non motor symptoms like hyposmia, rapid eye movement (REM) sleep behavior disorder (RBD) and a significant levodopa responsiveness. The fact that parkinsonism is required in both VP and PD and vascular changes are a common finding in older people including those with PD, explains the diagnostic dilemma, where a specific surrogate marker would be highly needed.

Subcortical and periventricular white matter hyperintensities (WMH) and lacunar infarctions are frequently observed in VP, correlating with the severity of motor symptoms, postural instability and falls (56, 62). While total or deep WMH are associated with aging and cerebrovascular risk factors, periventricular WMH appear more specifically linked to the severity of parkinsonism, particularly gait disturbances (55, 63, 64). Other cohorts, however, found no association between the burden of periventricular WMH or dopaminergic imaging findings and the motor severity (55, 65, 66), cognitive functions or depression (65). Moreover, 10%−30% of asymptomatic elderly individuals with vascular risk factors show subcortical lesions, further complicating the relationship between imaging findings and clinical symptoms (67).

In VP, ischemic damage disrupts motor circuits, particularly thalamocortical fibers to the supplementary motor area and cerebellar fibers controlling leg movement, due to lesions in the basal ganglia, thalamus, and subcortical white matter (52, 59, 62). Although some VP patients exhibit cell loss in the substantia nigra with a similar pattern to PD, the damage is generally less severe as shown in histopathological studies, suggesting that dopaminergic depletion is less prominent or absent in VP (52, 68, 69).

Dopaminergic imaging reveals distinct patterns that help differentiate VP from PD. Across larger cohorts, 60 to 70% of VP patients exhibit an abnormal striatal DAT uptake (56, 58, 63), but reductions are generally milder and more symmetric than in PD (53, 56, 58, 66, 70, 71). Quantitatively, age corrected striatal reduction averages 1.2% in VP compared to the 40.8% in PD (70). Compared with PD's posterior putamen-dominant asymmetry, VP often shows balanced involvement of both the anterior caudate and posterior putamen with a lower caudate/putamen (C/P) ratio (54–56, 58, 70). Striatal-to-background ratios (SNBRs) and striatal asymmetry indices (SAI) are useful metrics. Posterior putamen SNBRs at a cut-off of 1.85 yielded 100% sensitivity and 52.2% specificity for distinguishing PD from VP in one study (55). SAI also differentiates PD from VP, achieving 100% specificity for PD at a cut-off value of 14.08, though sensitivity is lower (54). Advanced techniques, such a machine learning approach with statistical parametric mapping, achieve over 90% accuracy for PD/VP discrimination (71). While abnormal dopaminergic imaging is highly predictive of PD, normal scan cannot reliably exclude PD due to its lower negative predictive value (12, 72). Approximately 30%−40% of VP patients, particularly in cases with WMH rather than basal ganglia involvement, show normal dopaminergic imaging indicating non-dopaminergic mechanisms such as disruptions in thalamocortical pathways (56, 58, 63). Conversely, asymptomatic individuals, such as those with NOTCH3 mutations, may show nigrostriatal denervation, reflecting a presymptomatic disease phase (73). The findings in dopaminergic imaging in VP are heterogeneous (see Table 3) (61, 74), with some studies showing a normal uptake in all VP patients (72), in contrast with the significant reduction observed in other studies (54, 66, 75).

**Table 3:** Summary of dopaminergic imaging studies in VP.

**Authors and year of publication**	**Nr patients**	**Radiotracer**	**Finding in dopaminergic imaging**	**Conclusions**
Antonini et al. (2012)	158 parkinsonism (82 PD, 76 VP)	^123^I-FP-CIT	DAT normal in 28 (36.8%) and reduced in 48 (63.2%); normal DAT associated with higher vascular load and poorer levodopa response	DAT status helps forecast dopaminergic therapy benefit; vascular burden modulates phenotype/response
Benítez-Rivero et al. (2013)	106 VP; 280 PD	^123^I-FP-CIT	32.5% of VP had normal scans; 100% of PD abnormal. VP showed higher uptake and lower asymmetry vs. PD	DAT SPECT plus clinical features improve VP vs. PD discrimination. ~50% of VP treated showed levodopa response
Contrafatto et al. (2011)	20 VP; 20 PD; 20 ET	^123^I-FP-CIT	SAI higher in PD than VP/ET. SAI >14.08 → 100% specificity, 50% sensitivity for PD vs. VP	Asymmetry-based indices can separate PD from VP in unclear cases
Funke et al. (2013)	15 VP; 15 PD	^123^I-FP-CIT	DAT deficit in 9/15 VP vs. 15/15 PD. VP: higher putaminal binding and lower asymmetry than PD	DAT loss is less pronounced and symmetric in VP; asymmetry and C/P ratios favor PD over VP
Gerschlager et al. (2002)	13 VP; 20 PD; 30 HC	^123^I-β-CIT	Striatal uptake was markedly reduced in PD (~40.8%) but near normal in VP (~1.2%). PD showed increased asymmetry and lower putamen/caudate ratios compared to VP	Presynaptic dopaminergic deficits typical of PD absent in most VP; DAT SPECT helps distinguish PD from VP
Huertas-Fernández et al. (2015)	80 VP; 164 PD	^123^I-FP-CIT (ROI + SPM)	Significant ROI/SPM differences; PD shows greater reduction in more-affected putamen and ipsilateral caudate	Machine-learning models achieved ~90% accuracy for VP vs. PD discrimination
Kim et al. (2019)	Three VP	^18^F-FP-CIT	Case 1: focal DAT decrease in left putamen from ipsilesional midbrain infarct Case 2: focal DAT defects in left caudate head/anterior putamen matching striatal infarct Case 3: early-phase PET showed focal thalamic infarct, late-phase DAT was normal bilaterally	Levodopa responsiveness varies with postsynaptic integrity; early-phase PET adds diagnostic value in VP beyond DAT binding alone
Lee et al. (2021)	23 VP; 23 PD; 31 HC	^18^F-FP-CIT	Despite visually normal scans, quantitative PET shows diffuse striatal DAT reduction in VP patients vs. controls; pattern distinct from PD	Periventricular WMH severity correlates with reduced DAT; quantitative analysis increases sensitivity in VP
Modreanu et al. (2020)	One cavernoma-related parkinsonism	^123^I-FP-CIT	Severe ipsilesional striatal reduction (caudate-predominant); mild contralateral decrease	Basal ganglia cavernoma mimicking PD; long-term non-progressive course; poor levodopa response
Navarro-Otano et al. (2014)	15 suspected VP; 15 PD; nine HC	^123^I-FP-CIT	VP showed heterogeneous DAT results: 7/15 normal, 3/15 PD-like, 5/15 with abnormalities differing from typical PD	DAT may be normal/atypical in VP; ancillary tests (MIBG, olfaction) useful when DAT is inconclusive
Ragno et al. (2013)	Five CADASIL with parkinsonism + three carriers without parkinsonism	^123^I-FP-CIT	Symmetric or asymmetric putaminal tracer reductions; two non-parkinsonian CADASIL also showed nigrostriatal denervation	Parkinsonism can occur late in CADASIL; imaging suggests vascular etiology; levodopa response generally poor
Solla et al. (2015)	One VP (midbrain infarct)	^123^I-FP-CIT	Focal reduction in ipsilesional putamen; ipsilateral caudate relatively spared; pattern distinct from typical PD lateralization	Hemiparkinsonism after left midbrain infarct; DAT pattern atypical for PD; moderate levodopa response
Tzen et al. (2001)	14 VP; 30 PD; 26 HC	^99^mTc-TRODAT-1	VP: uptake slightly lower than HC, not significant. PD: marked and asymmetrical reduction, more prominent in the putamen	TRODAT-1 differentiates PD from VP; VP usually lacks posterior putaminal loss
Vaamonde et al. (2007)	Five patients with striatal infarcts	^123^I-FP-CIT	Some DAT abnormalities discordant with lesion extent/location; two evolved consistent with underlying PD	Strategic infarcts can cause acute hemiparkinsonism; DAT may reveal coexistent PD
Vlaar et al. (2008)	248 parkinsonism (127 PD, 27 APS, 22 ET, 16 VP, five DIP)	^123^I-FP-CIT	FP-CIT odds ratio ~61 (95% CI 8–490) for distinguishing PD vs. VP	DAT SPECT is accurate for distinguishing PD from VP/DIP/ET; less useful for PD vs. APS
Zijlmans et al. (2007)	13 VP; 14 PD and 14 HC	^123^I-FP-CIT	VP showed reduced bilateral uptake vs. controls, but more symmetric than PD. Uptake reduction correlated with UPDRS, not with levodopa response	VP often shows mild, symmetric DAT reduction. Symmetry may aid differentiation from PD

This table summarizes published studies that investigate dopaminergic system involvement in patients with Vascular parkinsonism. For each study, the table lists the authors, year of publication, number of patients and tracer used, key findings in dopaminergic imaging and the study conclusions.

Dopaminergic imaging findings seem to correlate with other clinical markers of VP. Higher vascular load (periventricular and hemispheric WMH) has been reported more often in VP patients with normal DAT binding (63), whereas Lee et al. (55) found that reduced SNBRs in VP patients correlated with the severity of periventricular white matter hyperintensities. Imaging characteristics may differ between VPa and VPi, with higher asymmetry indices in cases of unilateral ischemic lesions affecting the nigrostriatal pathway (76).

**Figure 3:**
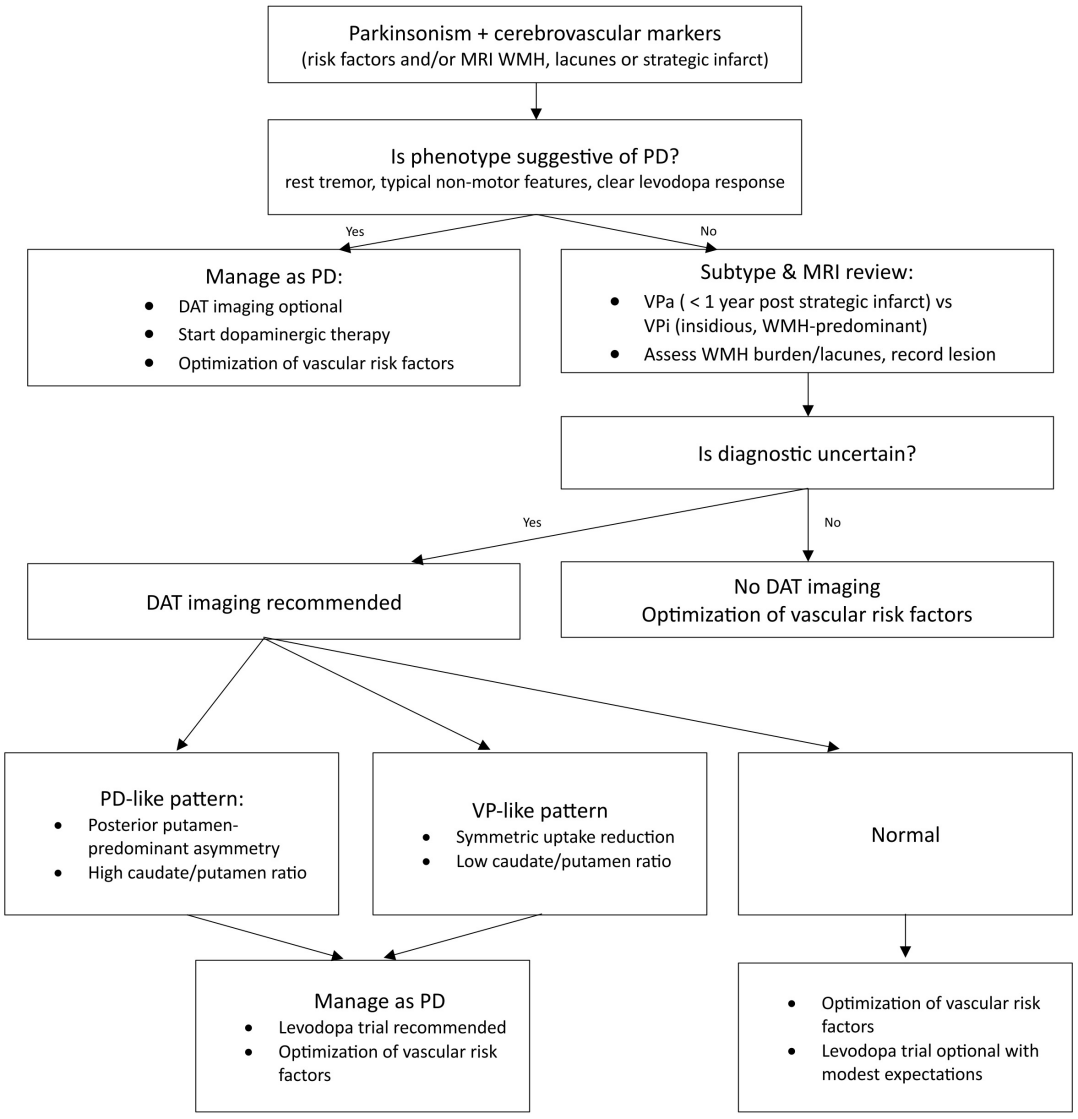
Diagnostic and treatment algorithm for patients with VP. This flowchart outlines the clinical assessment and imaging-guided management in VP. It summarizes clinical triage and MRI review for WMH, lacunes and strategic lesions. Obtain DAT imaging when diagnosis remains uncertain; interpret patterns as PD-like (levodopa trial recommended), VP-like (levodopa trial recommended) or normal (levodopa trial optional).

The relationship between dopaminergic imaging findings and levodopa response in VP is highly variable. Patients with normal striatal binding rarely benefit from dopaminergic therapy, with over 90% non-responder in one study with 76 VP patients (63), suggesting a non-dopaminergic cause for their symptoms (74, 77, 78). Among those with reduced DAT uptake, levodopa responsiveness varies widely, ranging from 20 to 50% (56, 57), nearly half may still fail to respond despite evidence of presynaptic dopaminergic deficits (63). Some cohorts report better motor improvement when dopaminergic imaging is abnormal (52, 53, 63, 74, 76, 78), whereas others find no clear levodopa benefit even in the presence of presynaptic deficits (53, 56, 71, 73, 75, 79). Overall, lesion topology seems influential: midbrain lesions disrupting the nigrostriatal pathway respond more often than striatal lesions or territorial infarctions (56, 74, 76). In practice, extent of vascular lesions, disease severity, and hypertension predict poor levodopa response more robustly than dopaminergic imaging alone (63). An imaging-guided pathway for selecting patients for DAT imaging and action on PD-like vs. VP-like patterns is provided in Figure 3.

While presynaptic dopaminergic imaging can aid distinguishing VP from PD, especially when combined with semi-quantitave metrics and clinical context, it cannot reliably confirm or exclude VP on its own. Given the frequent coexistence of VP and PD, assessing potential co-pathology is essential. The role of imaging in guiding levodopa therapy remains limited (61), as non-dopaminergic mechanisms contribute significantly to VP symptomatology (53, 79). However, in patients with a DAT deficit, we suggest a pragmatic levodopa trial, as some degree of responsiveness has been observed. Moreover, this trial can aid in distinguishing VP from PD, particularly in cases where postural instability and gait disorder (PIGD) subtype of PD is suspected. Given these complexities, VP management requires a broader approach, addressing vascular risk factors and tailoring treatment strategies based on the individual patient's imaging findings and clinical presentation (12). See Table 3 for study-level details and Figure 3 for a suggested imaging-guided algorithm.

### Dural arteriovenous fistula

Dural arteriovenous fistulas (DAVFs) are acquired vascular malformations with abnormal shunting between arteries and veins in the dura mater. The clinical symptoms vary depending on the location of the DAVF and on whether the lesion is solely dural or has a prominent drainage pattern (80).

First described by Matsuda et al. (81), the occurrence of parkinsonism with or without progressive cognitive dysfunction caused by DAVF has been rarely reported. Venous hypertension, which results from increased blood flow through the draining veins or from obstruction to drainage, has been associated with the neurological deficits (81–85). When the DAVF lead to a venous congestion of the galenic system, patients may present with parkinsonism (84, 85).

The decreased regional cerebral blood flow in the basal ganglia for patients with DAVF and parkinsonism has been demonstrated by a few authors (81, 84–88) but to our knowledge there are only two case reports of presynaptic dopaminergic imaging findings in DAVF-associated parkinsonism. Kim et al. (87) reported a patient with presynaptic dopaminergic deficit, which was not related to hemodynamic changes induced by DAVF. Previous symptoms with RBD, constipation and visual hallucinations, suggested underlying nigrostriatal degeneration unmasked or exacerbated by venous congestion. The patient's symptoms, except for the RBD, improved after DAVF occlusion without medication, suporting a hemodynamic aggravation of a pre-existing dopaminergic deficit (87). Kawasaki et al. (88) showed decreased dopamine transporter activity in the bilateral striatum in dopaminergic imaging with an improvement of the clinical symptoms after the treatment of the DAVF. Presynaptic dopaminergic imaging remains rarely documented, and no prevalence estimates are possible.

The response to dopaminergic treatment seems inconsistent in DAVF-associated parkinsonism. While some authors report at least a transient response to dopaminergic treatment (85, 86), others could not observe any benefit (84). The lack of response to dopamine has been interpreted for some authors as a sign of impaired postsynaptic striatal function due to venous congestion (88), but this remains hypothesis-level given the paucity of presynaptic data. In contrast, patients seem to have a clear benefit regarding their motor and cognitive symptoms after embolization of the DAVF, emphasizing the correlation between basal ganglia perfusion deficits and DAVF-related parkinsonism (84, 86, 87).

When DAVF-related parkinsonism is suspected, treat the fistula first. Reversing parkinsonism through successful DAVF treatment highlights the importance of addressing underlying vascular abnormalities to restore normal cerebral function and alleviate symptoms. Consider presynaptic dopaminergic imaging if the clinical picture suggests possible co-pathology (e.g., prodromal PD geatures) or if imaging would alter management.

## Infectious diseases

Many infectious diseases can potentially affect dopaminergic pathways and imaging. The ones mentioned below have some predilection for affecting striatal structures.

### Toxoplasmosis

Toxoplasma gondii can infect any nucleated cell, including within the central nervous system (CNS), crossing the blood-brain barrier during acute infection. During chronic infection, parasite cysts persist in the CNS, while being cleared from other organs (89). In mice, Toxoplasma cysts are found in various brain regions. However, it has a tropism for the basal ganglia, especially thalamus and striatum, as well as the amygdala, likely due to blood transport by the middle cerebral artery (89).

Clinically, movement disorders are uncommon in cerebral toxoplasmosis (CTx) and tend to be hyperkinetic, parkinsonism is rare. To our knowledge, there is only one case report documenting a (unilateral) presynaptic dopaminergic striatal deficit (90). Parkinsonism improved with levodopa after other treatments failed (90). CTx-related parkinsonism is likely due to direct damage to the substantia nigra rather than the basal ganglia, which would rather cause a postsynaptic dopaminergic deficit, as evidenced in this case by Magnetic resonance imaging (MRI) and dopaminergic imaging (90). Given the case-level nature of the evidence, the prevalence of presynaptic dopaminergic imaging abnormalities in CTx cannot be estimated, and presynaptic imaging should be considered selectively for atypical presentations or when a co-pathology is in the differential.

### HIV

Studies on HIV-related parkinsonism have shown variable prevalence rates and contributing factors. Mattos et al. (91) reported a 2.7% frequency of involuntary movements in patients with acquired immunodeficiency syndrome (AIDS), while half of them exhibited parkinsonian features. The most common causes of parkinsonism in these patients were HIV itself (12 patients), toxoplasmosis of the midbrain (3), and metoclopramide (3) (91). Recent observations suggest a decrease in HIV-related parkinsonism cases, possibly due to the introduction of highly active antiretroviral therapy (HAART) (91).

Presynaptic dopaminergic imaging evidence is limited and heterogeneous. A case study detailed a patient initially misdiagnosed with PD. Dopaminergic imaging showed bilaterally reduced putaminal uptake, with relatively preserved uptake in the caudate nucleus. The patient responded well to dopaminergic therapy and showed a complete clinical remission following HAART. A bilateral onset and rapid progression are common in HIV-associated parkinsonism but uncommon in PD. In this case the patient's presentation including levodopa-induced dyskinesia, which is uncommon in HIV-related cases, lead to the misdiagnosis of PD (92).

Another case, however, described a normal dopaminergic imaging in a patient experiencing rapid cognitive decline and parkinsonism, with only partial improvement after HAART, remaining care-dependent until death from pneumonia (93).

Because presynaptic imaging evidence in HIV-associated parkinsonism is limited to two case reports, no firm conclusions can be drawn.

### COVID

Although movement disorders in COVID-19 are rare, there have been a few reports of parkinsonism following severe COVID-19 infection. To our knowledge, only two studies have described the results of dopaminergic imaging in COVID-19 infection.

The mechanisms linking COVID-19 to parkinsonism remain unclear, but several hypotheses have been proposed. One possibility is direct viral invasion, as coronaviruses can access the CNS via the olfactory bulb, triggering neuroinflammation and microglial activation that may contribute to dopaminergic dysfunction (94). Supporting this, coronavirus antibodies have been detected in the CSF of PD patients, suggesting a potential immune-mediated mechanism (95). Alternatively, systemic inflammation and hypoxic-ischemic injury could exacerbate neurodegeneration. Severe COVID-19 leads to prolonged hypoxia, oxidative stress, and cytokine release, all of which have been linked to dopaminergic neuron vulnerability (96, 97). Infections also alter dopamine metabolism, reducing vesicular dopamine storage and increasing DAT activity, which may further impair striatal function (98). Another possibility is that COVID-19 may unmask preclinical PD in individuals with pre-existing but subclinical nigrostriatal deficits. Systemic infections have been associated with acute worsening of PD symptoms and in some cases, a faster disease progression (99). While this hypothesis remains speculative, it aligns with previous observations that systemic inflammation and metabolic stress can accelerate symptom onset in individuals already at risk for PD (100).

Dulski et al. reviewed 24 post-COVID-19 parkinsonism patients, noting that brain MRI was often normal while dopaminergic imaging showed impaired uptake in all eight patients in whom scans were performed. Levodopa responsiveness was reported in 12/15 treated patients; however, clinical improvement sometimes occurred without therapy or with immunomodulatory treatment, indicating a secondary etiology rather than primary neurodegeneration. Dopaminergic imaging patterns were asymmetric, paralleling findings from other viral infections linked to parkinsonism (97).

A case report described a 58-year-old man, who developed a hypokinetic-rigid syndrome with ocular abnormalities and opsoclonus following a severe SARS-CoV-2 infection. Dopaminergic imaging revealed an asymmetrical bilateral decrease in presynaptic dopamine uptake, particularly in the putamina. Significant spontaneous improvement in parkinsonian symptoms was observed without specific treatment. The authors discuss the similarities of this case with encephalitis lethargica, with similar acute symptoms like myoclonus and ocular motility disorders as well as fluctuating and transient changes in level of consciousness (96).

In the few post-COVID parkinsonism cases with presynaptic imaging, dopaminergic imaging confirms nigrostriatal dysfunction in those selected patients; however, the available evidence is limited to these case-level observations, so no firm conclusions can be drawn about prevalence, characteristic patterns, or prognosis. Further research is needed to determine whether these cases represent a distinct, virus-induced form of parkinsonism, the unmasking of pre-existing PD, or simply a coincidental onset of disease. The variability in clinical outcomes, including cases with spontaneous recovery, suggests that multiple mechanisms may be at play.

### Subacute sclerosing panencephalitis

Subacute Sclerosing Panencephalitis (SSPE) is a progressive, fatal encephalitis caused by a persistent mutated measles virus infection, usually emerging after a 4–10 year latency (101). The estimated incidence ranges between 6.5 and 11 per 100 000 measles cases annually (102), with the highest risk observed in individuals infected before age 5 (101, 102). Although the disease burden has declined due to widespread vaccination (101), cases persist in low-coverage regions (102), and reemergence has been noted in developed countries (103).

SSPE commonly begins during childhood or adolescence, but adult-onset cases have been reported, typically with atypical features (104). The clinical trajectory is classically divided into four stages (105):

- Stage I: behavioral and cognitive changes;
- Stage II: myoclonic jerks, seizures and progressive cognitive decline;
- Stage III: extrapyramidal symptoms;
- Stage IV: akinetic mutism and coma.

Movement disorders are present in up to 50% of the patients and are increasingly recognized as core features (106, 107). While myoclonus is most prominent (92% in some cohorts), other manifestations include dystonia (up to 48%), chorea (up to 14%), tremor (6.7%−39.1%), parkinsonism (5%−26%), ataxia (up to 18%), and rarely, tics and stereotypies (106, 107).

MRI abnormalities in SSPE may include periventricular white matter hyperintensities -more accentuated in the parieto-occipital cortex than the frontal cortex- cerebral atrophy, and basal ganglia involvement (104, 108).

Only limited data exist on dopaminergic imaging in SSPE. Hsieh et al. (109) reported combined FDG and FDOPA PET scans in a 19-year-old woman with stage II SSPE. FDG PET showed diffusely reduced cortical metabolism across both hemispheres, cerebellum, and thalami, while FDOPA PET showed preserved uptake in the striatum. The caudate showed slightly decreased glucose uptake relative to dopaminergic activity. The discrepancy suggests that dopaminergic terminals in the striatum are relatively spared early in the disease, despite extensive cortical and subcortical dysfunction. Singer et al. (104) described a 26-year-old woman with progressive visual loss, cognitive impairment and parkinsonism. [^18^F]fluoro-DOPA PET imaging showed preserved uptake in both caudate nuclei and the left putamen, but a slight reduction in the right putamen. This suggested relatively intact presynaptic dopaminergic function in early stages, despite profound extrapyramidal symptoms. The patient progressed to a state of myoclonus, retrocollis, and postural instability. At autopsy, extensive pathology was noted in cortical areas and the putamen, with notable neuronal loss in the substantia nigra—providing histological evidence for dopaminergic involvement in later disease stages.

To date, no curative therapy exists. Disease-modifying agents like intraventricular or intrathecal interferon-α and oral isoprinosine show mild effect at best (110). Symptomatic treatments like valproate, carbamazepine, clonazepam, or clobazam may alleviate myoclonus and movement disorders. However, most cases progress inexorably. Notably, adult-onset cases occasionally show a slower trajectory, transient stabilization or even spontaneous remission (104).

Dopaminergic imaging in SSPE is limited to anecdotal reports, yet provides valuable insights. The preservation of FDOPA uptake despite cortical hypometabolism aligns with clinical observations: hyperkinetic disorders, cognitive impairment and neuropsychiatric symptoms emerge earlier, while hypokinetic features like parkinsonism tend to appear later. This supports the hypothesis that basal ganglia circuits—particularly those involving dopaminergic terminals—may be relatively resilient until advanced disease stages. Given this selective vulnerability and only mild tracer uptake reduction in patients with clinical parkinsonism, further dopaminergic imaging studies could delineate dopaminergic dysfunction across disease stages and phenotypes. This would also clarify whether parkinsonism in SSPE results from presynaptic dopaminergic failure, or secondary network disruption.

### Japanese encephalitis

Japanese Encephalitis is a mosquito-borne viral CNS infection, which is endemic in Asia and western Pacific regions (111). The clinical spectrum ranges from asymptomatic infections to non-specific febrile illness, aseptic meningitis and acute encephalitis, which is the most common presentation (111, 112).

Even though most of the infections are clinically inapparent it is a severe, potentially lethal illness, with case fatality rate up to 30% (111). The disease course can be subdivided into a prodromal stage with fever, headache, fatigue, nausea and vomiting; the acute stage with changes in the mental status, confusion, encephalopathy, possible parkinsonian syndrome and/or upper and lower paralysis followed by the late stage with gradual recovery and possible persistence of CNS signs in up to 30%−50% of cases (111, 112). About one in four patients presents with a movement disorder, usually parkinsonism (113). The involvement of the basal ganglia has been described earlier from imaging studies with MRI (114, 115).

Our search of dopaminergic imaging retrieved only case-level evidence. There was one small case series with three patients aged 20–28 and two case reports of individuals aged 65–67. Despite the large age gap, the clinical presentation was similar with signs of acute infectious encephalitis and parkinsonian symptoms. A decreased striatal uptake in all patients was detected via ^123^I-FP-CIT and ^99^mTc-TRODAT-1. While Liao et al. and Tadokoro et al. showed an asymmetric striatal uptake, the patient observed by Lin et al. had a symmetric decreased striatal uptake (113, 116, 117). A longitudinal imaging study proposed an initial unilateral invasion of the thalamus with expansion throughout the disease course with the presentation of cytotoxic edema, as a possible explanation for these findings (118), although this remains hypothesis-level.

Available reports indicate acute striatal presynaptic reduction during encephalitic presentations. However, the limited evidence does not allow us to drawn any firm conclusion.

### Creutzfeldt-Jakob disease

Creutzfeldt-Jakob disease (CJD) is a rare and fatal prion disease, characterized by rapidly progressive neurodegeneration, with myoclonus, ataxia, rapid progressively dementia, akinetic mutism, and visual disturbances as presenting symptoms (14, 119, 120). Parkinsonism, dystonia and chorea are also possible clinical manifestations, strongly suggesting the involvement of the dopaminergic system (121). Parkinsonism can be an initial symptom of CJD, although it is relatively rare, sometimes leading to wrong diagnosis in the initial disease stage. However, the prevalence of extrapyramidal symptoms increases with longer disease duration and parkinsonism is frequently observed in the terminal stages of CJD, commonly in the form of akinetic mutism (14).

Multiple case studies have highlighted the relevance of dopaminergic imaging in CJD. A presynaptic dopaminergic deficit in CJD, typically with greater putaminal than caudate reduction, has been described by many authors, supporting the hypothesis of nigrostriatal dysfunction in this disease (121–127). This also holds true for familial CJD, where parkinsonism is more commonly observed compared to sporadic or variant forms of CJD (125, 128). Further supporting the link between CJD and nigrostriatal dysfunction, Vital et al. (14) conducted a neuropathological study revealing both pre- and post-synaptic cell loss in the nigrostriatal system of CJD patients, with the presynaptic dopaminergic loss being more accentuated in the cases of CJD presenting with parkinsonism than in those dominated by chorea or myoclonus. The loss of dopaminergic neurons in the substantia nigra correlated with the loss of neurons in the caudate and putamen, suggesting a parallel pre- and postsynaptic degeneration of the nigrostriatal pathway in CJD. Those cases underscore the importance of considering CJD in rapidly progressing atypical parkinsonism cases, as it may mimic an atypical parkinsonism, especially in the initial disease stage (123, 126, 128).

Park et al. (129) in contrast, presented a case of probable sporadic CJD with extrapyramidal symptoms as the main feature, where ^18^F-FP-CIT PET imaging showed no dopaminergic deficits. This finding has been supported by other authors, who described normal tracer uptake or only mildly decreased uptake on the caudate tail despite a marked parkinsonism in a patient with CJD (15, 130). This suggests that postsynaptic alterations may contribute to parkinsonism in CJD (129, 130).

Collectively, most of the published cases reveal significant dopaminergic deficits that correlate with clinical symptoms and neuropathological findings. Thus, while not diagnostic, it can provide supportive evidence of nigrostriatal dysfunction or indicate preserved presynaptic activity, suggesting a predominant postsynaptic pathology. Given the rapid progression and poor response to dopaminergic therapy, an early distinction from other parkinsonian syndromes can help guide diagnostic workup, patient counseling, and treatment decisions.

## Non-inherited metabolic diseases

Liver cirrhosis as well as diabetic uremic syndrome are both conditions with impaired metabolic processes and degradation products, who can lead to encephalopathy and impairment of the basal ganglia function (131, 132).

### Diabetic uremic syndrome

Diabetic uremic syndrome typically manifests as uremic encephalopathy with cortical involvement, resulting in seizures, asterixis, and myoclonus (132). A less common phenotype involves basal ganglia dysfunction, leading to parkinsonism, more unfrequently dyskinesia, chorea, and loss of consciousness (133). Pathophysiological factors include uremic neurotoxicity that impair mitochondrial function and disrupt the balance between excitatory and inhibitory amino acids, as well as microvascular and nutritive components (134).

Presynaptic dopaminergic imaging is limited to two case reports with divergent findings. Suzuki et al. (135) reported a 57-year-old man with diabetic renal failure, who presented with parkinsonism and loss of consciousness. His dopamine transporter imaging was normal, and his symptoms and MRI changes improved rapidly after hemodialysis, pointing to a reversible, non-nigrostriatal mechanism. Conversely, Ishii et al. (136) described a patient with sub-acute parkinsonian symptoms and a marked presynaptic dopaminergic impairment with no clinical improvement after hemodialysis or after levodopa administration. MRI showed vacuolated changes following focal necrosis, suggesting irreversible basal ganglia injury (136).

With only two presynaptic imaging cases, no firm conclusions can be drawn about prevalence or typical DAT patterns. Available reports suggest that normal DAT in this context aligns with dialysis-responsive and secondary parkinsonism.

### Liver cirrhosis

A subset of patients with chronic liver dysfunction may develop cirrhosis-related parkinsonism, regardless of the cause of liver failure (137, 138). Burkhard et al. (137) studied 51 patients eligible for liver transplantation over 1 year, finding that 11 (21.6%) had moderate to severe parkinsonism, with motor UPDRS ranging from 20.5 to 61. This condition differs from PD, presenting with rapidly evolving and symmetric akinetic-rigid syndrome, dysarthria, early gait and postural impairment, focal dystonia, and a prominent resting tremor (137, 139, 140).

Increased manganese (Mn) deposition, due to impaired Mn excretion via the bile duct (140), is a suggested cause, but it remains unclear why only a few patients develop parkinsonism since increased Mn is present in all cirrhotic patients (141). Chronic Mn accumulation is however not the only pathomechanism in cirrhotic disease. Synergistic effects of other toxic products like ammonium, neuroinflammation, and nitrosative stress are also contributing factors (131).

Yang et al. (142) reviewed 21 cases and proposed a classification into three categories: levodopa-resistant atypical parkinsonism without a dopaminergic deficit, coincidental PD with superimposed cirrhosis, and a subgroup of undetermined cases.

Pooling the presynaptic imaging reports we included (two case series, two case reports, one prospective cohort; total *n* = 17), 10/17 (~59%) showed preserved DAT/FDOPA uptake and typically a symmetric, rapidly evolving akinetic–rigid phenotype with limited levodopa benefit, consistent with secondary, cirrhosis-related parkinsonism, 6/17 (~35%) showed reduced presynaptic uptake, usually asymmetric with a posterior-putaminal (rostrocaudal) gradient, and levodopa responsiveness, compatible with coexistent degenerative PD/dual pathology, and 1/17 (~6%) had reduced uptake without levodopa response (undetermined) (139–143). Notably, MRI T1 basal-ganglia hyperintensity may occur regardless of presynaptic status. These proportions are exploratory and derived from case-level evidence, but they are practical for counseling: selective DAT SPECT/^18^F-DOPA PET helps set expectations (secondary vs. degenerative mechanisms) and identify the minority likely to benefit from dopaminergic therapy.

### Manganism

Mn is an essential trace metal primarily entering the body through diet. 98% of these Mn load is cleared by the liver, which explains the MRI and clinical findings resembling Mn intoxication in patients with chronic liver disease (144). A characteristic “manganism” phenotype is also seen with continuous consume of ephedrone (methcathinone), a CNS stimulant producing amphetamine-like effects, which is synthesized from pseudoephedrine, KMnO4, vinegar, and water (145–148). While exposure levels to Mn required to cause disease are unclear, serum Mn levels poorly correlate with neurological symptoms, with individual susceptibility influenced by factors like liver dysfunction, iron deficiency and genetic predisposition (149).

Mn-induced parkinsonism often presents with behavioral changes before parkinsonian features. The parkinsonism induced by Mn presents some features that helps differentiate this one from PD as a more symmetric presentation, kinetic tremor, dystonia, a characteristic gait known as “cock-walk” and early cognitive, balance, and speech issue. Some patients may show pyramidal signs. Patients are often younger at onset and unresponsive to levodopa therapy (145, 148–152). Parkinsonian features may progress after the cessation of Mn exposure (145, 150).

Mn toxicity damages GABAergic neurons in the globus pallidus, increases synaptic glutamate, reduces striatal dopamine, and disrupts iron regulation, causing oxidative stress and neuronal injury (145). MRI often reveals symmetrical T1 hyperintense signals in the globus pallidus, most severe in its medial part, the subthalamic nucleus and substantia nigra reticulata as well as putamen (145, 153, 154).

The dopaminergic imaging findings in manganism are heterogeneous. Although there are numerous studies documenting an intact nigrostriatal dopaminergic system (145–148, 151, 152), suggesting a predominant involvement of the postsynaptic dopaminergic pathway. There are a few reports of pathological dopaminergic imaging findings. The presynaptic dopamine deficit in those patients tends to be milder than in PD (155) and some patients may show an affection of the caudate, which is not so severely affected in PD (150).

Three non-exclusive explanations have been proposed for the presynaptic dopamine deficit observed in manganism: (i) an incidental Mn exposure in patients with PD, (ii) a degeneration in the presynaptic dopaminergic neurons caused by manganism, or (iii) Mn exposure as a risk factor for developing PD (156). Given the small, heterogeneous literature, no prevalence estimates can be made for presynaptic abnormalities or dual pathology.

In suspected manganism, preserved uptake in dopaminergic imaging is common and aligns with poor levodopa response and suggest a postsynaptic/network dysfunction. Abnormal DAT uptake should prompt consideration of PD co-pathology and may justify a levodopa trial with longitudinal follow-up. Dopaminergic imaging is therefore best used selectively to exclude degenerative nigrostriatal loss when clinical or MRI features suggest manganism, and to guide patient counseling about expected dopaminergic benefit.

### Osmotic demyelination syndrome

Osmotic demyelination syndrome (ODS) is a rare condition typically triggered by the rapid correction of hyponatremia, among other conditions, leading to central pontine myelinolysis (CPM) and extrapontine myelinolysis (EPM) due to degeneration and loss of oligodendrocytes (157, 158). Clinically, it presents with a biphasic course starting with encephalopathy or seizures due to hyponatremia, followed by mild improvement after correction of hyponatremia, and then a few days later deterioration due to myelinolysis (157, 158).

CPM primarily affects the corticobulbar and corticospinal tracts, resulting in dysarthria, dysphagia, quadriparesis, and potentially a locked-in syndrome, while EPM involves various brain regions leading to a wider spectrum of symptoms (158). MRI of patients with EPM show symmetric T2 hyperintensities and T1 hypointensities predominantly in the striatum and thalamus (159, 160), but the substantia nigra may also be affected (161). Some case reports have documented a putaminal atrophy in the chronic phase of ODS (162).

A study involving 11 patients revealed various movement disorders in ODS, including generalized dystonia (27.3%), parkinsonism (36.4%), or both (36.4%). Postural tremor was observed in 45.5% of the patients. Other movement disorders such as myoclonus, chorea, pseudochoreoathetosis, belly dancer dyskinesia, tics and ataxia can also be observed in ODS (157). The disruption of the frontal subcortical networks running through the striatum can lead to neurobehavioral symptoms (158). Patients commonly show features of both CPM and EPM (157, 158).

Evidence in ODS is limited to four case reports (*n* = 4). Across these, severe reduction in presynaptic striatal dopamine transporter density in patients with ODS-induced parkinsonism was demonstrated, suggesting damage to the nigrostriatal pathway (159–162), The asymmetry of clinical signs correlated well with asymmetric reduction in uptake of the radiotracer ligand (159–161). In two cases, recovery of clinical symptoms correlated with normalization of radiotracer uptake in follow-up studies, suggesting potential reversibility of nigrostriatal dysfunction (159, 162).

Cases have shown improvement with dopaminergic treatment (159–161), although responses can vary (157).

Overall, ODS can manifest as a complex array of neurological symptoms due to its diverse regional brain involvement, with movement disorders being prominent. These disorders can resolve (159, 160, 162), persist, or evolve over time (157, 161), necessitating tailored symptomatic treatment and close monitoring for long-term management (157, 159, 160, 162).

Although presynaptic evidence in ODS is limited to four single-patient reports, the findings are concordant. These case-level data suggest that presynaptic dysfunction in ODS-related parkinsonism is typically present and may be reversible, but numbers remain too small to infer prevalence or prognostic accuracy.

### Secondary chorea

Secondary chorea encompasses a group of hyperkinetic movement disorders arising from non-genetic systemic conditions, including infectious causes, drug-related chorea, autoimmune, metabolic or hematologic derangements (163). Although rare, these conditions provide a unique window into the vulnerability of the basal ganglia to systemic disturbances, highlighting complex interactions between vascular, metabolic, and neurotransmitter-related mechanisms.

In polycythemia vera (PV), chorea occurs in ~0.5%−5% of patients, with a striking female predominance (female:male ratio = 4:1) (164). Typically manifesting after age 50, the disorder presents with generalized chorea predominantly affecting the faciolingual and brachial muscles, often accompanied by muscular hypotonia (165). The hypothesized pathophysiology involves neostriatal hyperviscosity due to erythrocytosis, leading to venous stasis, impaired cerebral perfusion, and disturbed dopaminergic regulation (164, 166). Dopaminergic hyperactivity has been suggested, possibly enhanced by platelet-derived dopamine accumulation in the striatum or estrogen deficiency (164, 166).

Evidence in PV is based in one case report. Huang et al. (165) reported a patient with PV with moderate asymmetrical bilateral reductions in striatal tracer uptake on ^99^mTc-TRODAT-1 SPECT. Serial imaging showed a normalization of uptake and clinical improvement following therapeutic phlebotomies, suggesting a reversible presynaptic disturbance secondary to altered striatal perfusion or platelet-induced dopamine dysregulation rather than permanent neuronal loss. Conventional MRI is frequently unremarkable (165).

Diabetic chorea, also referred to as “diabetic striatopathy,” often presents as hemichorea-hemiballismus, more frequently in elderly women, with poorly controlled non-ketotic hyperglycemia (167). Pathologically, the disorder is characterized by selective striatal vulnerability to microvascular injury, including arteriolar wall thickening, capillary proliferation, erythrocyte extravasation, and patchy necrosis (168).

MRI consistently reveals T1-hyperintensity in the putamen, frequently unilateral and contralateral to the affected side, although bilateral cases have been described, that tend to resolve with glycemic control (167–170).

Presynaptic dopaminergic imaging in diabetic chorea is limited to two case reports. Sato et al. (169) demonstrated bilateral, right-predominant reductions in ^123^I-FP-CIT binding in the caudate and putamen using DAT-SPECT, with an asymmetry index of 10.7%, correlating with the contralateral dominance of chorea. This finding, along with reduced FDG uptake and perfusion in the same regions on PET and ^99^mTc-ECD SPECT, respectively, supports a model of focal nigrostriatal dysfunction in diabetic chorea. Notably, MIBG cardiac scintigraphy was normal, ruling out a generalized sympathetic denervation and underscoring the regional specificity of the damage (169). In another case, Belcastro et al. (170) reported strictly unilateral reductions in DAT binding in the putamen contralateral to symptoms, with a putamen/cortex ratio falling below the age-adjusted reference range. These results reinforce the presence of a localized presynaptic dopaminergic deficit, possibly resulting from energy failure in the metabolically vulnerable medium spiny neurons of the striatum (170).

Therapeutically, both conditions respond to the correction of the underlying systemic disturbance, phlebotomy in PV and glycemic normalization in diabetes (165, 170).

In conclusion, secondary chorea related to PV and diabetes mellitus represents a rare but informative window into the modulatory effects of systemic disorders on basal ganglia circuitry. While both disorders exhibit characteristic imaging abnormalities, the underlying mechanisms diverge, ranging from reversible perfusion-related dysfunction in PV to microvascular injury in diabetes. These insights underscore the importance of considering metabolic and hematologic etiologies in adult-onset chorea and the role of dopaminergic imaging in these disorders.

## Autoimmune receptor encephalitis

Autoimmune encephalitis are due to numerous antineuronal antibodies, which, by affecting basal ganglia, are prone to induce movement disorders. Some antibodies are associated with specific movement patterns and concomitant symptoms, such as IgLON5 (171, 172).

Indeed, patients with IgLON5 antibodies typically develop a sleep disorder along with bulbar symptoms, parkinsonism, gait abnormalities, oculomotor disturbances, and sometimes cognitive decline, mimicking a PSP clinical presentation (173, 174). The physiopathology is believed to be both autoimmune and neurodegenerative, involving neuronal tauopathy (97, 99).

To date, dopaminergic imaging findings in IgLON5 have only been described in a few case reports. Most IgLON5 cases with dopaminergic imaging show bilateral striatal reduction compatible with nigrostriatal involvement (175–178). Phenotypes include one patient with signs of LBD (175), two with a PSP-like syndrome (176, 177) and another with a corticobasal degeneration-like syndrome (178). Notably, Fuseya et al. (178) reported clinical improvement after treatment with intravenous methylprednisolone pulse therapy for 5 days followed by a 5-day intravenous immunoglobulin (IVIG) course with significant increased striatal tracer uptake on follow-up, suggesting partial reversibility of presynaptic dysfunction in at least some patients. In contrast, Bruggemann et al. (179) described a case with orolingual and limb dystonia, executive dysfunction, progressive gait impairment, abnormal eye movements and unremarkable dopaminergic imaging findings. In this case, treatment with immunosuppressive and symptomatic therapy provided only mild improvement. Given the absence of characteristic parasomnias and of IgLON5 antibodies in CSF, the attribution to IgLON5 disease in the serum-only positive case is uncertain, and alternative etiologies should be considered.

Beyond IgLON5, three additional case reports link other antibodies to presynaptic abnormalities (172, 180, 181). Otsuka et al. (181) showed an improvement of dopaminergic imaging after treatment in an 84-year-old man with Hashimoto-encephalopathy and Anti-NAE-Antibodies who clinically mimicked multiple system atrophy with left dominant limb and truncal ataxia, slurred speech and urinary disturbance, without apparent parkinsonism.

Similarly, Endres et al. (172) reported a 63-year-old female with anti-glycine receptor antibodies, who presented with personality changes, cognitive decline and parkinsonian syndrome over a 3-year course. Dopaminergic imaging revealed a severe bilateral reduction of striatal dopamine transporter availability. While immunotherapy led to a stabilization of symptoms, levodopa treatment allowed a slight improvement of mood and rigidity. A possible overlap with neurodegenerative PSP was considered by the authors.

A 67-year-old Japanese woman with anti-recoverin antibodies presented with fever, somnolence, resting tremor and rigidity. Dopaminergic imaging revealed reduced radiotracer uptake in the basal ganglia. Treatment with levodopa could not alleviate symptoms, but high-dose IVIG did (180).

These cases illustrate the complex interplay between autoimmune and neurodegenerative processes (172, 180). While some patients respond to immunotherapy, others exhibit persistent dopaminergic deficits, raising questions about cross-reactivity between antibodies and basal ganglia structures (180) as well as the possibility of underlying neurodegeneration (172). The spectrum of antibody-associated basal ganglia encephalitis remains vast, with many causes still unknown (182). As Kitazaki et al. (180) note, dopaminergic imaging reports in autoimmune basal ganglia encephalitis remain scarce, underscoring the need for further research.

## Discussion

Dopaminergic imaging is well-established for diagnosing and differentiating PD from other conditions such as essential tremor or drug-induced parkinsonism. Numerous studies have demonstrated its accuracy in documenting degeneration of the nigrostriatal pathway at the presynaptic level in degenerative parkinsonism (12). However, the modality's limitations, particularly its modest specificity among degenerative syndromes and incomplete separation from some acquired disorders restricts its potential as a definitive biomarker and underscore the need to complement imaging findings with additional biomarkers. In this regard, emerging evidence suggests that combining dopaminergic imaging with molecular assays such as RT-QuIC for alpha-synuclein aggregation or other dementia markers can offer a more comprehensive diagnostic approach (24).

This review synthesizes how presynaptic dopaminergic imaging contributes to the diagnosis and management of several acquired diseases affecting the basal ganglia. Three themes emerge. First, presynaptic dopaminergic imaging may help discriminating degenerative from secondary mechanisms when clinical and other paraclinical findings are equivocal. Second, in several acquired disorders, it can track reversibility—or lack thereof- after treating the underlying cause. Third, in some conditions it shapes counseling about dopaminergic therapy and prognosis by revealing whether nigrostriatal dysfunction is likely.

The first important point is whether presynaptic signal is reduced or preserved, because this maps to mechanism and management. In iNPH, above half of the imaged patients in our review showed a presynaptic deficit, with cohort-level rates spanning roughly 31%−91% and a pooled proportion of 86/167 (~51.5%) across studies reporting binary outcomes, supporting genuine nigrostriatal involvement in a sizeable fraction (26–30). In HT, aggregating 15 reports (30 patients), 17/30 (~57%) showed reduced presynaptic uptake on DAT imaging (37–46), while 13/31 (~43%) had preserved/normal binding (47–49). In VP, presynaptic loss is also common but heterogeneous: ~60–70% of patients across series exhibit reduced uptake, whereas 30–40%, particularly those with diffuse white-matter disease (VPi), do not (56, 58, 63). By contrast, in cirrhosis-related parkinsonism, pooled case-level data suggests presynaptic reduction in only about one-third, with the remainder preserved (139–143). Manganism stands at the other end of the spectrum: presynaptic integrity is typically preserved, and PD-like loss is uncommon and inconsistently reported (145–148, 150–152, 155). For ODS, all four published single-patient reports demonstrated reduced striatal binding, consistent with nigrostriatal involvement in that setting (159–162). Infectious and autoimmune conditions sit between these poles: in the limited samples with imaging, Japanese encephalitis and post-COVID parkinsonism generally showed reduced uptake in the patients who were actually scanned (96, 97, 113, 116, 117); and autoimmune encephalitis (e.g., IgLON5, anti-NAE, anti-GlyR, anti-recoverin) shows reduced presynaptic signal in almost all reported cases (172, 175–178, 180, 181). Finally, in CJD, presynaptic reduction is predominant across case reports, though normal scans have also been described despite parkinsonism, implying that postsynaptic or network mechanisms can occasionally dominate the clinical picture (121–130). For a summary of presynaptic dopaminergic uptake reduction across the different acquired conditions see Table 4.

**Table 4:** Summary of presynaptic dopaminergic uptake across acquired conditions.

**Condition**	**Total imaged (*n*)**	**Reduced uptake *n* (%)**	**Preserved uptake *n* (%)**	**Strength of evidence**
Idiopathic NPH	167	86 (51%)	81 (49%)	One randomized clinical study, cross-sectional comparative studies and case reports/series
Holmes tremor	30	17 (57%)	13 (43%)	Case reports and case series
Vascular parkinsonism	253	151 (60%)	102 (40%)	Cross-sectional comparative studies, one cohort and case reports/series
Dural arteriovenous fistula	2	2 (100%)	0	Case reports
Toxoplasmosis	1	1 (100%)	0	Case report
HIV	2	1 (50%)	1 (50%)	Case reports
COVID	9	9 (100%)	0	Case reports/serie
Subacute sclerosing panencephalitis	2	0	2 (100%)	Case reports
Japanese encephalitis	5	5 (100%)	0	Case reports
Creutzfeldt–Jakob disease	11	8 (73%)	3 (27%)	Case reports
Diabetic uremic syndrome	2	1 (50%)	1 (50%)	Case reports
Cirrhosis-related parkinsonism	17	7 (41%)	10 (59%)	Case reports/series and one cohort
Manganism	36	28 (78%)	8 (22%)	Case report/series
Osmotic demyelination syndrome	4	4 (100%)	0	Case reports
Secondary chorea	3	3 (100%)	0	Case reports
Autoimmune receptor encephalitis	8	7 (88%)	1 (12%)	Case reports

Values show the proportion of abnormal (reduced) presynaptic uptake among patients who underwent presynaptic dopaminergic imaging in the studies included in this review. Only studies reporting an exact per-study count or percentage of pathological vs. normal scans were included in the pooled proportions; studies providing group-level quantitative metrics only without per-patient classification were excluded from this table and summarized qualitatively in the text. Rows based on small case-series or single cases are indicated, and should be interpreted with caution.

Notably, parkinsonism may arise with normal DAT when pathology is postsynaptic, or when symptoms reflect network/disconnection rather than primary nigrostriatal loss. Further, DAT's negative predictive value is limited. Early or mild degenerative loss can be visually normal, and subtle/symmetric change is missed without semi-quantification (12, 72). Clinically, preserved DAT with parkinsonism should shift attention toward postsynaptic/network mechanisms and non-dopaminergic or cause-directed therapies, while keeping room for re-assessment if the phenotype evolves.

Beyond presence or absence, where presynaptic loss occurs, its topography and symmetry, often carries actionable signal. A posterior-putaminal-predominant, asymmetric reduction with a greater AI and a low caudate/putamen ratio is the canonical PD-like pattern and shifts probability toward degeneration in vascular or NPH-like uncertainty (28, 54, 55). By contrast, iNPH tends to show more symmetric, caudate-weighted uptake reductions and a higher C/P ratio than PD when presynaptic dopaminergic imaging is abnormal (18, 30), helping differentiated a mechanical dysfunction from classic PD physiology. In vascular parkinsonism, reductions, when present, are typically milder and more symmetric, with relatively less posterior-putamen emphasis and a higher C/P ratio than PD (54, 56, 58, 70). Quantitative parameters may help us differentiate VP from PD (54, 55). In liver cirrhosis, a PD-like posterior-putaminal gradient is consider for some authors as a sign of an underlying PD as dual pathology, whereas a preserved binding aligns with secondary parkinsonism (139–143). In conditions where only case-level data exist, pattern-level interferences remain tentative; here, semi-quantification, as C/P ratio and asymmetry indices, should be routine to avoid overinterpreting visual reads while we await larger datasets.

A distinctive contribution of presynaptic imaging in acquired disorders is that it functions as a biological stress test of the nigrostriatal pathway: when the cause is treated, improvement or normalization of dopaminergic imaging argues for a reversible circuit failure, whereas a stable PD-like deficit points to fixed degeneration or co-pathology. This distinction is clearest where interventions directly relieve the precipitating factor. In iNPH, a subset of patients shows higher striatal binding after CSF diversion, paralleling gait gains, evidence that mechanical distortion can depress presynaptic function that later recovers, while other patients progress toward synuclein/tau phenotypes with persistently abnormal scans, consistent with underlying neurodegeneration (21, 23, 31). Similar reversibility appears during recovery from osmotic demyelination (159, 162), and after phlebotomy for PV chorea (165), where rises in presynaptic signal track clinical improvement. In autoimmune encephalitis, isolated reports show increased striatal uptake after steroids/IVIG, again suggesting a treatable, immune-mediated component in some cases (178, 181).

The clinical yield of presynaptic imaging is highest when the answer will alter management rather than merely label a phenotype. In vascular parkinsonism, iNPH, or HT, imaging may help predict which patients may benefit from a levodopa trial. While the role of levodopa trial in NPH has been debated, there are some reports of a clinical improvement under dopaminergic therapies (18, 32). We propose that in cases with abnormal dopaminergic imaging results, a trial of dopaminergic therapy should be considered before a more invasive therapy take place. In iNPH presynaptic dopaminergic imaging may also play a role in predicting the outcome after VPS, as some authors have observed a poorer outcome after VPS in patients with a pathological dopaminergic imaging (26, 27, 33). Therapeutic implications in HT revolve around understanding the degree of dopaminergic dysfunction, which can predict levodopa responsiveness (38, 40, 43, 46, 48, 50) and may support selection of the DBS target (subthalamic nucleus (STN), Gpi/PTT vs. VIM). However, while a variety of pharmacological treatments and surgical interventions like DBS have shown some effectiveness, levodopa remains a cornerstone, particularly in cases where nigrostriatal involvement is evident. Our proposed therapeutic algorithm (Figure 2) involving levodopa supplementation, especially in patients with dopaminergic denervation on imaging, could optimize outcomes in select HT patients. However, variability in response reflects the underlying heterogeneity of HT's pathophysiology, with some patients no benefiting from levodopa besides the documented affection of the nigrostriatal pathway (37, 41, 44). In VP patients normal dopaminergic imaging is a significant predictor of a negative response to levodopa treatment (63). Whereas in almost half of VP patients with abnormal dopaminergic imaging, levodopa treatment does not show a major clinical improvement (56, 57, 63), in VP patients with abnormal scan, levodopa therapy may still be of benefit (52, 53, 63, 74, 76, 78) (See Figure 3).

When interpreting these findings, we should keep in mind some limitations. The literature remains dominated by case reports and small series outside VP and iNPH, limiting precision in estimating the prevalence and patterns of DAT abnormalities. Methodological heterogeneity across tracers, acquisition protocols, reference regions, and thresholds further constrains cross-study comparisons. Our review was limited to presynaptic imaging by design, which sharpens its focus but excludes complementary insights from postsynaptic or metabolic modalities. Study selection was performed by a single reviewer, and no formal risk-of-bias assessment was conducted. Prospective, multi-center cohorts with harmonized acquisition and semi-quantitative analysis, coupled with longitudinal outcomes (levodopa responsiveness, disease evolution after etiology-directed therapy), are needed to define the prognostic value of presynaptic imaging across acquired conditions. Integration with fluid biomarkers (e.g., NfL, Alzheimer-spectrum markers) and α-synuclein seeding assays may further refine co-pathology detection and treatment selection, especially in mixed presentations.

## Conclusion

Dopaminergic imaging has expanded beyond its traditional role in diagnosing PD to encompass a broad spectrum of acquired neurological disorders. While its use should be judicious to ensure cost-effectiveness, dopaminergic imaging remains a valuable diagnostic aid, particularly for patients with ambiguous clinical presentations. Its ability to detect both reversible and irreversible changes in dopamine transporter activity offers clinicians valuable insights into disease mechanisms and therapeutic responses. In conditions such as HT, NPH, VP, and liver cirrhosis, dopaminergic imaging not only aids in diagnosis but may also predict treatment decisions in certain cases, particularly regarding the use of levodopa. As research continues to evolve, the integration of imaging with pathological findings will likely refine diagnostic algorithms, particularly in differentiating between neurodegenerative and non-neurodegenerative parkinsonism. Dopaminergic imaging thus stands as a critical tool in both clinical practice and research, with significant implications for personalized patient care.
